# Characterization of H_2_S QEPAS detection in methane-based gas leaks dispersed into environment

**DOI:** 10.1016/j.pacs.2022.100438

**Published:** 2022-12-13

**Authors:** Mariagrazia Olivieri, Giansergio Menduni, Marilena Giglio, Angelo Sampaolo, Pietro Patimisco, Hongpeng Wu, Lei Dong, Vincenzo Spagnolo

**Affiliations:** a PolySense Lab - Dipartimento Interateneo di Fisica, Politecnico and University of Bari, Via Amendola 173, Bari, Italy; b State Key Laboratory of Quantum Optics and Quantum Optics Devices, Institute of Laser Spectroscopy, Shanxi University, Taiyuan 030006, China

**Keywords:** Quartz-enhanced photoacoustic spectroscopy, Hydrogen sulfide, Methane, Hazardous gas leaks detection, Gas and oil wells, Environmental monitoring

## Abstract

The increase in fatal accidents and chronic illnesses caused by hydrogen sulfide (H_2_S) exposure occurring in various workplaces is pushing the development of sensing systems for continuous and in-field monitoring of this hazardous gas. We report here on the design and realization of a Near-IR quartz-enhanced photoacoustic sensor (QEPAS) for H_2_S leaks detection. H_2_S QEPAS signal was measured in matrixes containing up to 1 % of methane (CH_4_) and nitrogen (N_2_) which were chosen as the laboratory model environment for leakages from oil and gas wells or various industrial processes where H_2_S and CH_4_ can leak simultaneously. An investigation of the influence of CH_4_ on H_2_S relaxation and photoacoustic generation was proposed in this work and the sensor performances were carefully assessed with respect to CH_4_ content in the mixture. We demonstrated the high selectivity, with no cross talk between H_2_S, H_2_O and CH_4_ absorption lines, high sensitivity, and fast response time of the developed sensor, achieving a minimum detection limit (MDL) of 2.5 ppm for H_2_S with 2 s lock-in integration time. The employed 2.6 µm laser allowed us to employ the sensor also for CH_4_ detection, achieving an MDL of 85 ppm. The realized QEPAS sensor lends itself to the development of a portable and compact device for industrial monitoring.

## 1 Introduction

Trace gas detection is among the main issues in industrial and urban area environmental monitoring, atmospheric science, and medical applications. Hydrogen sulfide (H_2_S) is one of the most challenging gases to detect since it is hazardous, corrosive, and flammable even at very low concentrations. The Occupational Safety and Health Administration (OSHA, US Department of Labor) fixed at 20 part per million (ppm) the concentration limit for H_2_S breathing, for an eight-hour period, with a maximum peak exposure of 50 ppm for ten minutes [1]. The National Institute for Occupational Safety and Health determined an IMDH (Immediately Dangerous to Life and Health) concentration limit of 100 ppm for H_2_S. The typical rotten eggs smell, perceptible starting from concentrations of few ppm, disappears at 100 ppm and olfactory paralysis and eye irritation could occur. Exposures to concentrations higher than 500 ppm are fatal [2].

Human activity represents the main source of H_2_S global emission. This gas is largely produced in coke ovens, sewerage, tanneries, pulp and paper industries [3]. H_2_S concentrations ranging from few ppms to percents are found in crude oil and natural gas (NG) reservoirs [4]. A recent study on releases of methane (CH_4_) and H_2_S from active, abandoned and marginally producing oil and gas wells in Ontario (Canada), classifies these emissions as major risks for human and ecosystem health [5]. In addition, leakages from NG transmission pipelines systems typically occur. In their last survey, the European Gas Pipeline Incidents Data Group (EGIP) reported that the major contributions for gas pipelines leakages arise from external interferences, corrosion, construction defects or material failure, and natural disasters such as earthquakes [6]. H_2_S is also found as a by-product in NG processing plants during raw NG purification and in petroleum refiners, where H_2_S is produced during hydrodesulfurization of petroleum feedstocks and fuels [7].

According to the OSHA, approximately 100 accidents involving H_2_S exposure (70 % of which were fatal) occurred over the last two decades in a variety of workplaces in USA, including oil and gas wells, gas and petroleum plants, sewer lines, and tanks [1]. In addition, the release of H_2_S impose serious threats also for the environments and residents surrounding the leak sources [8], [9]. Thereby, the development of sensors capable of real-time and continuous monitoring of H_2_S leaks dispersed into environment has become an urgent need. Beyond the fast response and sensitivity, the high selectivity represents the main characteristic to fulfil, provided the high probability to identify an H_2_S leak source that is strictly related to a hydrocarbon-based matrix, mainly composed of CH_4_.

Many techniques have been used for H_2_S detection. Gas chromatography was largely exploited, but despite the high sensitivity and precision, the need for a sample preparation consisting in multiple steps makes this technique not suitable for real-time measurement and detection of short-term concentration variations [10]. Chemical sensors, such as those based on semiconductor metal-oxides [11] or electrochemical sensing techniques [12], [13] have been demonstrated to provide high sensitivity and real-time monitoring. However, their performances are strongly affected by environmental conditions, such as temperature and humidity levels.

Optical sensors represent an effective solution, providing both high sensitivity and high selectivity through laser excitation of gas absorption transitions in the infrared range. Mid-IR Quantum Cascade Lasers (QCLs) combined with multipass cells have been employed for H_2_S detection at trace level, demonstrating their applicability for industrial processes aimed at petrochemical environments [14], [15]. Chen et al. [16] obtained a minimum detection limit (MDL) of 670 part per billion (ppb) for 2 s averaging time, by means of off-axis integrated cavity output spectroscopy combined with a distributed-feedback (DFB) diode laser emitting around 1.57 µm. A similar telecom fiber-coupled laser and two resonant photoacoustic cells were employed in photoacoustic spectroscopy (PAS) based-systems developed for industrial applications [17], [18]. An MDL of 6 ppm was achieved for 10 s integration time [17], whereas long averaging time (∼30 min) were required to reach detection limits as low as 500 ppb [18]. PAS basic principle consists in detecting sound waves induced by gas non-radiative energy relaxation as consequence of infrared modulated light absorption. Quartz-enhanced photoacoustic spectroscopy (QEPAS) represents an evolution of the PAS approach and exploits a quartz tuning fork (QTF) to transduce the acoustic wave into an electric signal. The use of QTFs avoids the need for a photodetector, which is mandatory for multipass cells and cavity-based spectroscopy [19]. Reduced dimensions, high sensitivity and fast response time make QEPAS a perfect candidate for realization of compact and portable sensors to be employed for on-site measurements [20], [21], [22]. Recently, PAS and QEPAS sensors were developed both for hydrocarbon detection at high concentration or at trace-level [23], [24], biomedical applications [25] and detection of dangerous and toxic gases [26], [27].

H_2_S strongest absorption lies in the THz spectral region. Sampaolo et al. [28] demonstrated a QEPAS sensor for H_2_S detection, exploiting a liquid-nitrogen-cooled THz QCL operating in pulsed mode. By targeting a line-strength of 5.53∙10^-20^ cm/mol, an MDL of 2.5 ppm at 300 ms lock-in integration time has been achieved. However, poor quality THz beams, lack of commercially available laser sources, costs and dimensions create serious limits to the implementation of portable sensors. In the Mid-IR spectral range, QCL sources are commercially available with continuous wave output power > 100 mW, but the average absorption linestrength is almost two order of magnitude lower with respect to the THz spectral range [29], [30]. Nevertheless, the record in detection limit was achieved in the Mid-IR. Indeed, Helman at al. [29] reached an MDL of 492 ppb for an integration time of 1 s, by exciting a line-strength of 7.77⋅10^−22^ cm/mol with an off-beam QEPAS configuration and a continuous wave external cavity QCL (∼160 mW optical power). In the Near-IR spectral range, low cost, low weight, and low power consumption DFB diode lasers have been exploited for H_2_S detection. An absorption line at 1.57 µm having a linestrength of 1.15⋅10^−23^ cm/mol was excited in a QEPAS sensor realized by Kosterev et al. [31] to achieve an MDL of 10 ppm with 35 mW laser power available for PA generation. A sub-ppm MDL was reached employing a similar laser source relying on a erbium-doped fiber amplifier to boost the optical power up to ∼1.5 W [32], [33]. Viciani et al. [34] achieved a 4 ppm detection limit at 1 s integration time, exploiting a diode laser emitting around 2.6 µm, with an optical power of only 3 mW, but exploiting a line-strength one order of magnitude stronger with respect to the sensor reported in ref [31].

However, none of these research works has ever directly dealt with the main issue afflicting all the H_2_S optical sensors. i.e., the almost unavoidable spectral interference between H_2_S and CH_4_. This problem is absent in the THz range but is a crucial issue when operating in Near-IR and Mid-IR spectral regions. Moreover, for photoacoustic sensing, it is essential to study the response of the sensor system when the gas matrix fluctuates. This requires a detailed analysis of the energetic levels involved in the non-radiative energy relaxation processes to the experimental characterization of the photoacoustic generation and detection. This specific research, focused on H_2_S photoacoustic detection in a CH_4_-based matrix, has never been carried out as well, so far.

In this work we address these two open issues, demonstrating a Near-IR-QEPAS sensor for H_2_S detection providing fast response, high selectivity, and sensitivity. Mixtures of H_2_S traces in nitrogen (N_2_) or air with CH_4_ concentrations up to 1 % were obtained to simulate natural gas leaks dispersed into environment. The influence of CH_4_ on H_2_S detection in terms of PA generation and interference effect was investigated. Moreover, the employed 2.6 µm DFB laser allowed us to target a Near-IR CH_4_ transition as well, with the possibility of achieving a sequential H_2_S/CH_4_ detection.

## 2 H_2_S and CH_4_ relaxation paths

**Fig. 1:**
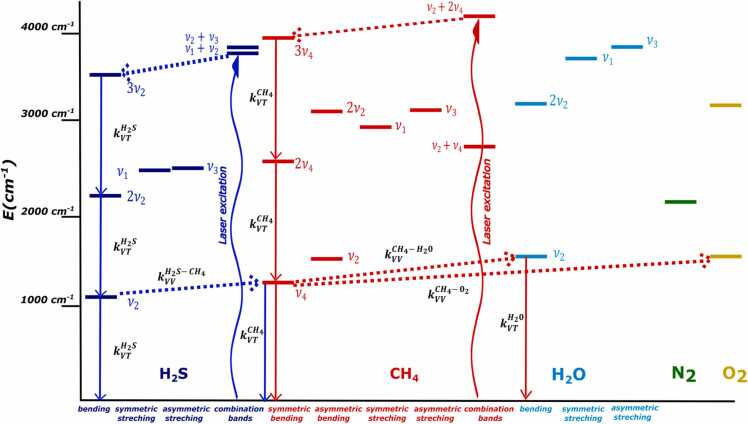
Energy level diagram showing the laser excitation and the relaxation paths of CH_4_ (red arrows) and H_2_S (blue arrows) molecules. The red and blue curved arrows are the laser excitations of CH_4_ and H_2_S energy levels, whereas the solids and dashed arrows represent V-T and V-V processes, respectively*.* (For interpretation of the references to color in this figure legend, the reader is referred to the web version of this article.)

When a gas sample, characterized by a known composition, is dispersed into the environment, the photoacoustic detection of one (or many) molecules within the gas leak requires a detailed investigation of the influence of gas sample matrix, diluted and mixed with the main atmospheric components. The 2.6 µm Near-IR region was identified as spectral-wise suitable for QEPAS detection of H_2_S and CH_4_. A schematic representation of the infrared energy levels diagram of H_2_S, CH_4_ and the main atmospheric gas species (N_2_, oxygen O_2_, and water vapor H_2_O) is represented in Fig. 1. For illustration purposes, each spectral band is represented by an energy level placed at the band center. In the region chosen for H_2_S/CH_4_ excitation, CH_4_ spectrum consists of a bending band 3ν_4_ and a combination band ν_2_ + 2ν_4_, creating a cluster of interacting states, i.e. a polyad, at higher energy levels (not shown in the figure) [35]. When one of these states is excited, the vibrational energy is suddenly (in the ns range) transferred to the lower state 3ν_4_ of the octad. Then, CH_4_ relaxation occurs via vibrational to translational (V-T) processes: a bending quantum ν_4_ is lost by collisions with the other molecules in their ground states. The overall process is characterized by a collisional rate given by [36]:

(1) $$k_{\mathrm{VT}}^{{\mathrm{CH}}_{4}}\;=P\sum_{i}C^{i}k_{\mathrm{VT}}^{{\mathrm{CH}}_{4}\;-M_{i}}$$

where P is the pressure, C^i^ is the concentration of the collisional partners and $k_{\mathrm{VT}}^{{\mathrm{CH}}_{4}\;-M_{i}}$ is the V-T relaxation rate associated to collisions between the excited molecule $\text{C}{\text{H}}_{4}^{\text{*}}$ and the molecule M_i_ in its ground state. Because of its hydrogen bonds and low moment of inertia, H_2_O acts as a V-T relaxation promoter for CH_4_. This effect was investigated in several works [37], [38], [39]. Moreover H_2_O bending mode could be excited via a vibrational-vibrational (V-V) process: the transferred energy is released as kinetic energy for CH_4_ PA generation due to the fast H_2_O V-T relaxation via self-collisions and collisions with N_2_ [40], [41]. Similarly, energy relaxation of CH_4_ is accompanied by a resonant excitation of O_2_ first excited level. However O_2_ relaxation rates are much lower than QEPAS modulation frequencies thus the transferred energy is lost for PA generation [42], [43]**.**

The V-V processes rate will depend upon the gas pressure and collisional partner concentration M_i_ according to:

(2) $$k_{\mathrm{VV}}=PC^{i}k_{\mathrm{VV}}^{{\mathrm{CH}}_{4}-M_{i}}$$

The V-T and V-V relaxation rates of excited $CH_{4}^{*}$ with the main atmospheric components are reported in Table 1.

**Table 1:** V-T relaxation rates of the n^th^ CH_4_ vibrational state with the main collisional partners in standard air. The reactions in bold represent the V-V processes.

	k (s^-1^Torr^-1^)	Ref
$C{\text{H}}_{4}^{\text{*}}\left(\text{n}{\text{ν}}_{4}\right)+\text{C}{\text{H}}_{4}\to \text{C}{\text{H}}_{4}^{\text{*}}\left({\left(\text{n}-1\right)\text{ν}}_{4}\right)+\text{C}{\text{H}}_{4}$	1.32*10^3^	[42]
$\text{C}{\text{H}}_{4}^{\text{*}}\left(\text{n}{\text{ν}}_{4}\right)+{\text{H}}_{2}\text{O}\to \text{C}{\text{H}}_{4}^{\text{*}}\left({\left(\text{n}-1\right)\text{ν}}_{4}\right)+{\text{H}}_{2}0$	1.05*10^5^	[37]
$\text{C}{\text{H}}_{4}^{\text{*}}\left(\text{n}{\text{ν}}_{4}\right)+{\text{N}}_{2}\to \text{C}{\text{H}}_{4}^{\text{*}}\left({\left(\text{n}-1\right)\text{ν}}_{4}\right)+{\text{N}}_{2}$	1.32*10^5^	[42]
$\text{C}{\text{H}}_{4}^{\text{*}}\left(\text{n}{\text{ν}}_{4}\right)+{\text{O}}_{2}\to \text{C}{\text{H}}_{4}^{\text{*}}\left({\left(\text{n}-1\right)\text{ν}}_{4}\right)+{\text{O}}_{2}$	1.71*10^3^	[42]
$CH_{4}^{*}\left(\nu_{4}\right)+O_{2}\to CH_{4}+O_{2}^{*}$	4.34*10^**3**^	[43]
$CH_{4}^{*}(\nu_{4})+H_{2}O\to CH_{4}+H_{2}O^{*}(\nu_{2})$	2.60*10^**4**^	[40]

Analogously H_2_S excitation and relaxation are examined. H_2_S spectra is characterized by a triad composed of the bending mode 3ν_2_ and two combination bands (ν_1_ + ν_2_, ν_2_ + ν_3_) [44], [45]. However, to our knowledge, no studies reporting on H_2_S V-V and V-T relaxation paths and the associated collisional rates are available in literature, so far. Nonetheless the following hypothesis could be done:

• Laser excitation of one of H_2_S states of the triad is followed by energy transfer to 3ν_2_ band, which is the lowest state of the triad. H_2_S V-T relaxation results in the loss of the lowest energy quantum ν_2_ and can be described by a formula similar to Eq. (1).
• H_2_S shows similar vibrational de-excitation velocity compared to H_2_O due to their similar chemical properties. Thus, the effects of H_2_O as a V-T promoter for H_2_S relaxation should be almost negligible [46]. Indeed, Viciani et al. [34] observed this effect in the 2.6 µm Near-IR spectral region for up to 2 % H_2_O concentration.
• A V-V exchange is energetically possible between H_2_S*(ν_2_) and CH_4_ since the energy difference (Δν = 128 cm^-1^) is less than the thermal energy (k_B_T ∼ 207 cm^-1^) [47]. V-V exchanges with other molecules are unlikely due to higher energy gap between the involved vibrational bands.

## 3 Experimental apparatus

**Fig. 2:**
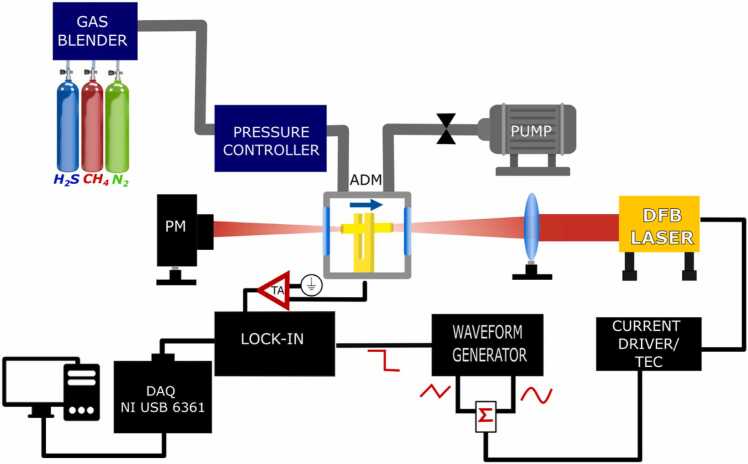
Schematic of the experimental apparatus. ADM-acoustic detection module, DFB- distributed feedback laser, TEC-thermoelectric cooler, PM-power meter.

The schematic of the experimental setup is shown in Fig. 2.

The laser source is a DFB diode laser emitting around 2638 nm with an output power up to 12 mW. A thermoelectric cooler (TEC) and a current driver (CD) allow setting the laser temperature and providing current for the laser diode, respectively. The laser light is focused through an acoustic detection module (ADM) by means of a plano-convex Si lens, with a focal length of 75 mm. The ADM consists of a gas cell equipped with an inlet and an outlet window, allowing the gas mixture to flow through the airtight chamber containing a spectrophone, composed of a T-shaped QTF and in-plane acoustic resonator (AR) tubes [48]. The spectrophone has a resonance frequency f_0_ = 12,458.52 Hz and quality factor Q = 32,140 at P = 100 Torr in a mixture containing pure N_2_. A voltage ramp and a sinusoidal dither are applied to the laser source to finely tune the laser emission wavelength and modulate the laser light at the half of the QTF fundamental resonance mode f_0_/2, respectively. Both signals are provided by a TEKTRONIC AFG 31,000 waveform generator. The piezoelectric current was converted into an electrical signal by a transimpedance amplifier (TA) and the f_0_ component was detected by a lock-in amplifier (2 f-Wavelength Modulation). A data acquisition card (National Instrument USB 6361) and a LabVIEW-based software are used to acquire the demodulated signal. The gas handling system is composed of an MCQ Instrument Gas Blender GB-100, used to manage the flow rate for three gas channels and produce the desired gas mixture. Pure N_2_ was used as carrier gas. A Nafion humidifier (PermSelect PDMSXA) was placed downstream the gas mixer to humidify the samples (not shown in the figure), fixing the H_2_O concentration for all measurements at 1 % of absolute humidity. Relative humidity and temperature within the gas line were measured by an IST AG HYT 271 sensor positioned near the ADM (not shown in the figure). An MKS type 649 pressure controller/flow meter, in combination with a needle valve and a pump allowed fixing the gas pressure and monitoring the flow rate inside the gas line. The gas flow rate was fixed at 50 sccm, with a 1 % of accuracy of the flow setpoint of each channel provided by the instrument datasheet.

## 4 Sensor performance for H_2_S and CH_4_ detection

### 4.1 Preliminary characterization

By tuning the laser temperature, the Near-IR spectral region from 3788 cm^-1^ to 3795 cm^-1^ can be investigated. With the aim of pursuing a sequential detection of CH_4_ and H_2_S, the detection scheme requires the laser temperature to be set at T = 20 °C to excite the CH_4_ absorption line located at 3791.67 cm^-1^, with a line strength of 4.41·10^-26^ cm/molecule for 1000 ppm of CH_4_. Then, the laser temperature is set at T = 15 °C to target the H_2_S absorption line at 3793.24 cm^-1^ with a line strength of 1.47·10^-26^ cm/molecule for 10 ppm of H_2_S [49]. The optical power available for PA generation, in correspondence of both the absorption lines selected, was measured to be ∼ 7 mW. QEPAS sensor response was also studied in terms of gas pressure and modulation amplitude and optimized for H_2_S detection.

**Fig. 3:**
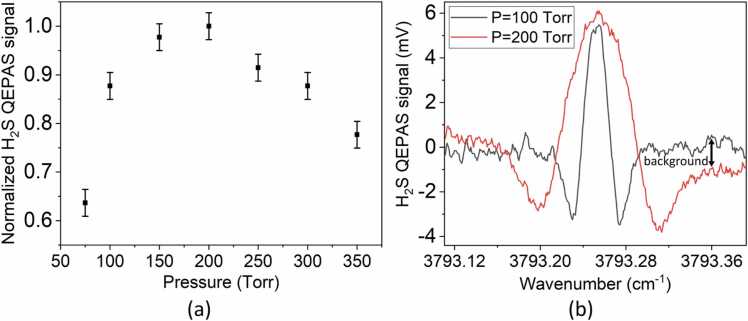
a) Normalized H_2_S QEPAS peak signal as a function of gas pressure. The data refer to a gas mixture of 150 ppm of H_2_S in N_2_. b) Spectra of 150 ppm of H_2_S in N_2_ recorded at 100 Torr and 200 Torr at 100 ms integration time. The non-zero background absorption at P = 200 Torr is marked by the black arrow.

Fig. 3a reports the H_2_S QEPAS peak signal as a function of the gas pressure for a gas target concentration of 150 ppm diluted in N_2_. The pressure maximizing H_2_S QEPAS signal was experimentally found to be 200 Torr. In Fig. 3b, a comparison between QEPAS spectra of a mixture composed of 150 ppm H_2_S in N_2_ acquired at 100 Torr and 200 Torr, respectively, with 100 ms integration time, are shown.

**Fig. 4:**
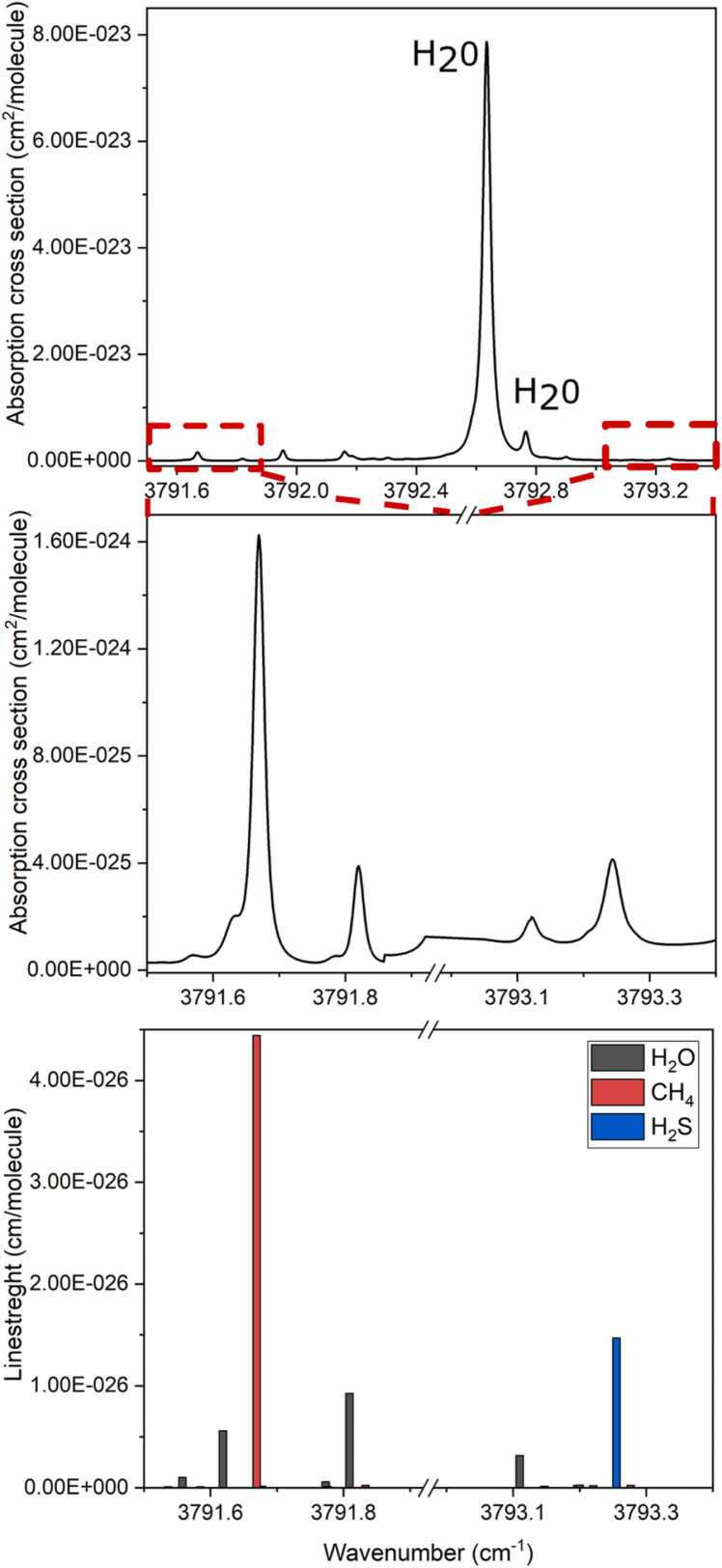
Absorption cross section of a mixture of 10 ppm of H_2_S, 1000 ppm of CH_4_ and air (1.19 % H_2_O, 20.90 % O_2_, 77.87 % N_2_ and traces of other gases) at P = 100 Torr and T = 296 K. Upper panel: simulation in the spectral region 3791.5–3793.4 cm^-1^. Central panel: enlarged view around the selected H_2_S and CH_4_ absorption lines. Lower panel: stick spectrum of H_2_O, H_2_S and CH_4_ analytes in the same ranges of the central panel.

It can be easily noted that at 200 Torr, a non-zero background absorption arises with respect to the noise level at 100 Torr. This is due to the interference of a nearby absorption line of H_2_O. This effect is levelled off at P = 100 Torr, with only a ∼ 10 % loss in the QEPAS signal. As a result, all measurements for both analytes were carried out at P = 100 Torr, with an optimum amplitude modulation of 20 mV. HITRAN database was used to simulate the absorption cross section of CH_4_-based gas leak, containing H_2_S, dispersed in air and analyzed at a pressure of 100 Torr. The simulated spectrum, shown in Fig. 4, is related to 1000 ppm of CH_4_ and 10 ppm of H_2_S, mixed with a standard air mixture containing typically 1.19 % H_2_O, 20.90 % O_2_, 77.87 % N_2_ and traces of other gases [49].

The simulation clearly shows the spectral scenario to deal with: at 100 Torr, H_2_S and CH_4_ do not interfere each other and H_2_O is the only absorber potentially interfering with the two target molecules. Once experimentally verified that, at the selected operating pressure**,** the pressure broadening of the H_2_O line at 3792.6 cm^-1^ (Fig. 4, upper panel) is small enough to not influence the H_2_S signal background, the possible H_2_O interference on CH_4_ detection must now be excluded. Thereby, it was verified that almost all the nearby H_2_O lines were under the sensitivity of our QEPAS sensor. The only detectable H_2_O absorption feature at 3791.8 cm^-1^ did not affect CH_4_ line shape at the selected working pressure.

Following these preliminary characterizations, the most appropriate integration time τ for sensor calibration was chosen. Based on a possible further development and engineering of this QEPAS prototype as H_2_S in-situ and real-time leak detector, a trade-off must be found between fast-response time and high sensitivity. We experimentally verified that 2 s integration time and 4 s acquisition time allowed us to unambiguously detect H_2_S QEPAS signal with respect to noise level in the few ppm scale, providing a signal to noise ratio (SNR) of ∼ 4 for a 10 ppm H_2_S concentration in N_2_, as it will be demonstrated in the following section.

### 4.2 H_2_S and CH_4_ calibration in N_2_

**Fig. 5:**
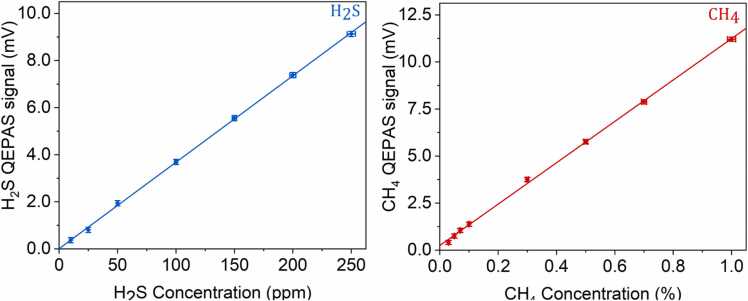
QEPAS peak signal versus CH_4_ (left panel) and H_2_S (right panel) concentration acquired at 2 s integration time and P = 100 Torr. The solid lines represent the best linear fits.

Firstly, the sensor calibration for H_2_S and CH_4_ detection was carried out in a concentration range typical of a CH_4_-based leak containing H_2_S, dispersed into the environment. CH_4_ concentration was varied from 0.03 % to 1 % using a certified mixture of 1 % CH_4_ in N_2_ and N_2_ as carrier gas. Similarly, we analyzed the QEPAS response for H_2_S detection from 10 ppm to 250 ppm using a certified mixture of 250 ppm of H_2_S in N_2._ The calibration curves, consisting in the 2 f-QEPAS signal peak values acquired at different concentrations, with a 2 s integration time, are shown in Fig. 5. A linear trend was verified for both analytes with a linearity coefficient of 0.0368 mV/ppm and 0.0011 mV/ppm for H_2_S and CH_4_, respectively.

**Fig. 6:**
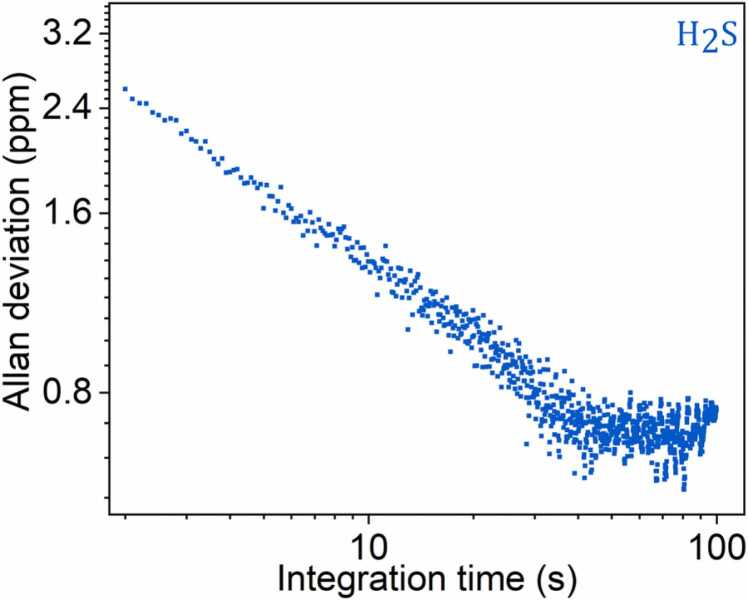
Allan deviation of H_2_S QEPAS signal as a function of integration time. At 2 s integration time, the calculated MDL is 2.5 ppm.

In order to investigate how the detection limits of the sensor improve as a function of the integration time, an Allan-Werle deviation analysis of H_2_S QEPAS signal was performed [50]. A 2-hour long acquisition (0.1 s lock-in integration time and 0.3 s acquisition time) was carried out in N_2_ at P = 100 Torr and at a laser current fixed far from gas absorption. The results are shown in Fig. 6: MDL is plotted as a function of integration time starting from 2 s integration time with MDL equal to 2.5 ppm. The NNEA for H_2_S detection was found equal to 8.8 * 10^-9^ cm^.1^W/Hz^1/2^. H_2_S detection limit can be further improved to the sub-ppm scale by increasing the integration time up to 30 s, but such sensitivity and response time levels are not functional for real time detection*. By performing a similiar analysis for CH_4,_ a MDL of 85 ppm was calculated for a 2 s integration time.*

### 4.3 Influence of CH_4_ on H_2_S detection

**Fig. 7:**
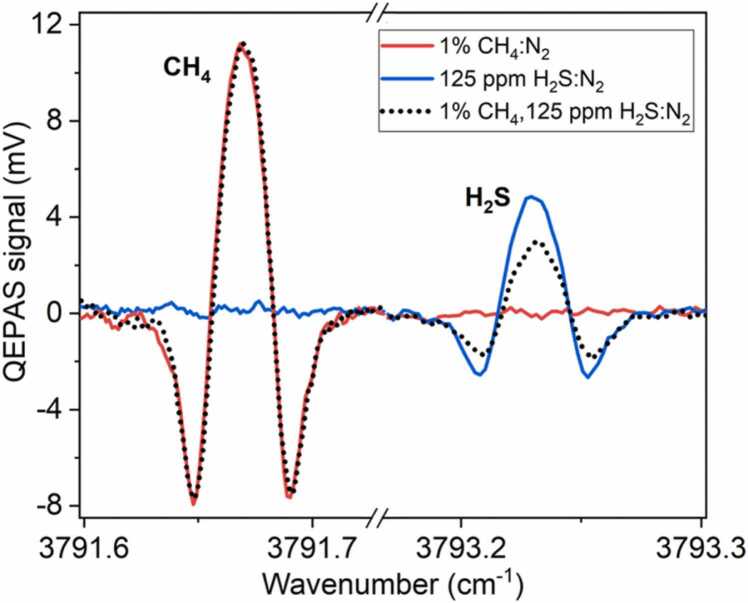
2 f CH_4_ and H_2_S QEPAS spectra measured for three mixtures: 1 % CH_4_ in N_2_,125 ppm H_2_S in N_2_ and 1 % CH_4_ and 125 ppm H_2_S in N_2_.

The sensor performances for detection of both analytes in the same mixture were analyzed. In Fig. 7, the measured QEPAS signal for a N_2_-based mixture of 1 % CH_4_ and 125 ppm H_2_S, obtained diluting a 10 % certified concentration, is compared to the QEPAS spectrum of the single analytes diluted in N_2_.

It is evident that H_2_S traces do not influence the CH_4_ QEPAS signal neither in terms of interference effect nor as a V-T promoter. Conversely, a ∼ 38 % reduction of the H_2_S peak signal was observed when increasing the CH_4_ concentration in the mixture from 0 % to 1 %. When a spectrophone is employed in a QEPAS sensor for gas detection, several factors can affect the detection of a given target molecule, such as H_2_S in this case [51].

#### 4.3.1 Influence of gas density on QTF resonance properties

QTF quality factor was measured by electrically exciting the QTF around the expected resonance frequency. By performing a Lorentzian fit, the QTF quality factor measured for a mixture composed of 1 % CH_4_ and 125 ppm H_2_S in N_2_ was found equal to 32,933, differing by less than ∼3 % with respect to the Q measured with no CH_4_ in the mixture (32,140). This difference is also lower than the uncertainty on Q-factor value calculated from the fitting errors. Thereby this effect can be also neglected. These variations in the matrix composition didn’t affect the resonance frequency as well, shifting from 12,458.54 Hz to 12,458.52 Hz. The experimental results were also compared to theoretical calculations. The Q-factor related to fluid damping can be derived by the model developed by Hosaka et al. which is an approximation for vibrating rectangular prongs oscillating in a viscous gas matrix [52]. The applicability of this model to QTFs vibrating at the fundamental and overtone mode was experimentally demonstrated [53], [54]. In addition, the model was validated for T-shaped QTFs, like the one employed in this work [55]. Based on this model, the damping contribute to the overall quality factor can be theoretically evaluated as follows:

(3) $$Q_{\mathrm{gas}}=\frac{4{\rho \mathrm{Tw}}^{2}f_{o}}{3\mu_{\mathrm{gas}}w+\frac{3}{4}w^{2}\sqrt{\left(4{\pi \rho }_{\mathrm{gas}}\;\mu_{\mathrm{gas}}f_{o}\right)}}$$

where $f_{o}=12458.52\mathrm{Hz}$ is the QTF resonance frequency, w=0.25mm is the crystal width, T=1.4mm is the prong thickness and $\rho =2650\;\mathrm{kg}/m^{3}$ is the quartz density. $\rho_{\mathrm{gas}}=\;M_{\mathrm{gas}}\;P/\mathrm{RT}$ and $\mu_{\mathrm{gas}}$ are the gas density and the dynamic viscosity of the mixture, respectively. Both $M_{\mathrm{gas}}$ and $\mu_{\mathrm{gas}}$ are calculated as a sum of each gas species molar mass and viscosity, respectively, weighted by their concentration. By using Eq. (3) it can be evaluated that a $Q_{\mathrm{gas}}$variation larger than 3 % is obtained for CH_4_ concentrations higher than 8 %, which is well above the typical concentration range for leaks detection.

#### 4.3.2 Influence of sound speed on AR tubes’ signal enhancement

Gas mixture composition could modify the speed of sound, causing a detuning between acoustic resonance of the resonator tubes and that of the QTF. Indeed, the effective tube length for the fundamental resonance mode of resonator tubes depends upon the sound velocity v_s_ according to [56]:

(4) $$\text{l}=\frac{{\text{v}}_{\text{s}}}{2{\text{f}}_{\text{o}}}-\frac{16\text{d}}{3\text{π}}$$

where f_0_ is the QTF fundamental resonance frequency and d is the internal tubes diameter (1.59 mm). Evaluating v_s_ through the ideal gas sound velocity [31] a negligible variation of ∼ 26 µm in the optimum tube length was calculated when increasing the CH_4_ concentration in the mixture from 0 % to 1 %. Significant decreases in the QEPAS peak signal has been observed only for tube length variations larger than 1 mm with respect to the optimal tube length [48]. Thereby also in this case the effect on the QEPAS signal is negligible.

#### 4.3.3 CH_4_ influence on H_2_S relaxation dynamics

**Fig. 8:**
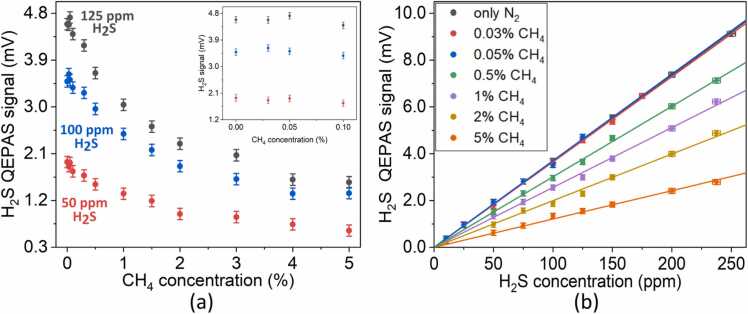
(a) H_2_S QEPAS signal as a function of CH_4_ concentration. The data refer to H_2_S concentrations of 50 ppm (red dots), 100 ppm (blue dots) and 125 ppm (black dots). The inset shows an enlarged view in the 0.03–0.1 % range of CH_4_ concentration (b) H_2_S calibration in mixtures containing both CH_4_ and N_2._ (For interpretation of the references to color in this figure legend, the reader is referred to the web version of this article.)

It remains one last effect to investigate, i.e., the V-V energy transfer (see Fig. 1) from H_2_S to CH_4_ molecules, which can negatively affect the H_2_S photoacoustic generation due to a retarded energy relaxation. We extended the CH_4_ concentration range up to 5 %, to investigate the influence of the matrix effect on H_2_S relaxation*.* Based on the previous calculations, the acoustic QTF response was considered flat for all the investigated mixtures. This was also confirmed experimentally. The QEPAS peak signals measured for 50 ppm (red dots),100 ppm (blue dots) and 125 ppm (black dots) H_2_S concentrations are plotted as a function of CH_4_ concentration in Fig. 8a. H_2_S signal was experimentally found to be not affected by CH_4_ contamination in the carrier gas up to 0.1 % concentration. This is clear from the inset of Fig. 8a, where the fluctuation of the H_2_S peak signal mean value over 4 acquisitions, observed in the range 0.03–0.01 %, fall within the 1-σ noise error bars calculated for each CH_4_ concentration (0.1 mV). This is also confirmed by comparing in Fig. 7b the nearly perfect overlap between the H_2_S calibration curves obtained in N_2_ with those measured for mixtures containing CH_4_ concentrations of 300 ppm and 500 ppm. The linear fit coefficients, their uncertainty and the calculated MDLs are reported in Table 2. Thereby, the obtained results demonstrate that the presence of CH_4_ molecules up to per thousand range in the N_2_-based matrix do not significantly modify the H_2_S V-T relaxation rate, which thus occurs primarily thorough collisions with gas components at tens of per cents concentration, namely N_2_.

**Table 2:** Slopes and MDL of H_2_S calibrations obtained by using mixtures of N_2_ and CH_4_ as carrier gas.

	Only N_2_	0.03 % CH_4_	0.05 % CH_4_	0.5 % CH_4_	1 % CH_4_	2 % CH_4_	5 % CH_4_
**Slope (mV/ppm)**	0.0368 ± 0.0001	0.0365 ± 0.0003	0.0370 ± 0.0005	0.0301 ± 0.0002	0.0256 ± 0.0003	0.0199 ± 0.0003	0.0121 ± 0.0002
**MDL (ppm)**	2.5	2.5	2.5	3	3.7	4.7	7.8

As CH_4_ concentration increases above the per thousand scale, H_2_S QEPAS signal drops down reaching a plateau for concentrations > 4 %, corresponding to a peak value reduction of ∼ 70 %. Exponential decrease of PAS signal cannot be explained by simply assuming V-T relaxation but are clear evidence of the presence of V-V processes resulting in energy transfer to slower relaxation channels [42], [57], [58]. Based on the discussion presented in the first section, a V-V exchange from H_2_S* (ν_2_) to CH_4_ is likely to happen due to their similar energy. If CH_4_ concentration is sufficiently high, the rate associated with this process given by $C_{{\mathrm{CH}}_{4}}{\mathrm{Pk}}_{\mathrm{VV}}^{H_{2}S-{\mathrm{CH}}_{4}}$, becomes comparable to the rate characterizing the V-T process $k_{\mathrm{VT}}^{H_{2}S}$(see Eq. (1)). However, the vibrational energy transferred to CH_4_ molecules is not efficiently converted into translational energy, and thus available for photoacoustic generation, due to CH_4_ slow relaxation times with respect to water-like molecules, such as H_2_S. The observed plateau corresponds to a saturation of the number of collisions occurring between H_2_S and CH_4_.

In principle, a phase shift should be directly related to the above-mentioned V-T, V-V processes. [42], [57], [58]. Nevertheless, the relatively low operating pressure of 100 Torr used for all the measurements didn’t allow an unambiguous interpretation of the phase data. In fact, the phase shift in the photoacoustic signal can be calculated as Ф(τ) = arctan (2πfτ), where f is the modulation frequency and τ is the non-radiative relaxation time. Provided that $\tau^{-1}=P\sum_{i}C_{i}k_{i}$[42], the capability of the system in recognizing phase shifts due to variations in concentration of the collisional partners strongly decreases at lower pressures, where the slope of the Ф(τ) function is lower as well.

From this investigation, it follows that the H_2_S calibration curve must be necessarily adapted to the CH_4_ concentration to properly interpret the QEPAS signals and accurately retrieve the H_2_S concentration, when the CH_4_ component in the gas matrix exceeds 1000 ppm.

Thereby, several calibration curves have been extracted for different CH_4_ concentration, up to 5 % and the obtained results are reported in Fig. 8b and Table 2. An H_2_S sensor MDL lower than 4 ppm was calculated for CH_4_ concentration up to 1 % and worsens to ∼ 8 ppm for 5 % CH_4_ concentration, where CH_4_ quenching effect is saturated. It is worth to notice that the obtained detection limits at 2 s integration time are below the OHSA's long exposure limit.

**Fig. 9:**
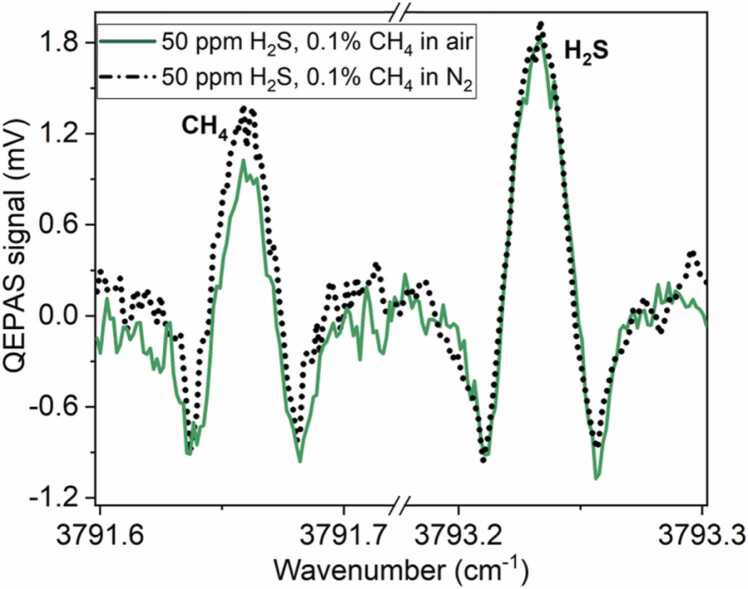
2 f CH_4_ and H_2_S QEPAS spectra measured for two mixtures: 50 ppm H_2_S, 0.1 % CH_4_ and N_2_. 50 ppm H_2_S, 0.1 % CH_4_ and air.

The applicability of H_2_S/CH_4_ detection in a standard air matrix was also investigated. With this purpose, we acquired QEPAS spectra for mixtures obtained by replacing N_2_ with standard air (20 % O_2_, 1 % H_2_O and 79 % N_2_) as carrier gas. Fig. 9 shows a comparison between the spectra obtained for a mixture of 1000 ppm of CH_4_ and 50 ppm of H_2_S diluted in N_2_ and in standard air, respectively. A 30 % reduction of the CH_4_ QEPAS signal was observed. This experimental evidence, together with the resonance properties of the resonator remaining unchanged also in standard air, points in the direction of a V-V exchange occurring between excited CH_4_ and O_2_ molecules, as explained in the first section. The energy transferred to O_2_ is thus expected to be substantially lost due to the slow V-T relaxation time (0.63 ms) compared to one period of laser modulation (0.08 ms) resulting in a lower CH_4_ signal. Consequently, CH_4_ signal interpretation should take into account the presence of O_2_ and of the fluctuations in H_2_O concentration [58]. On the contrary, H_2_S peak remained unaffected by the matrix variation, showing that no efficient V-T or V-V exchange can occur with O_2_ molecules, neither with H_2_O. Thus, the calibrations obtained for H_2_S detection in a matrix containing CH_4_ up to per thousands, H_2_O (∼ 1 %) and N_2_ are still valid when the gas matrix is represented by standard air, since O_2_ at the ∼ 20 % level does not affect the photoacoustic generation.

## 5 Conclusions

We presented a Near-IR-QEPAS sensor capable of a fast-response sensing of H_2_S trace-gas, within a matrix containing N_2_ and CH_4_ up to few percent. Spectral window of operation and detection parameters, such as gas sample pressure and modulation amplitude, were identified in order to avoid spectral interference among H_2_O, CH_4_ and H_2_S absorption lines. The high selectivity provided by the sensing system allowed us to focus on the analysis of H_2_S photoacoustic detection non-linearities arising from the interaction with the gas matrix. Firstly, the complete scheme of the energy levels involved was evaluated to qualitatively identify the possible pattern of V-T and V-V energy transfers. Then, a systematic characterization of the H_2_S signal cross-correlations with other components was carried out. Indeed, we demonstrated that V-V energy transfer from H_2_S to CH_4_ for CH_4_ concentrations larger than 1000 ppm degrades H_2_S QEPAS signal. Nonetheless, we demonstrated that the sensor detection limit for H_2_S detection at 2 s integration time was well below the OHSA’s long exposure limit (20 ppm) in all the investigated gas matrices. MDL was calculated as low as 4 ppm for CH_4_ concentration ≤ 1 % in the matrix, which can be assumed as a reasonable reference concentration representing relatively small natural gas leaks dispersed into environment. We validated the sensor for H_2_S detection in a standard air-based matrix, demonstrating the sensor potentiality for on-field and real-time measurements deployment.

As a future development of this experimental investigation, the presented measurements can be repeated at higher pressures to obtain a more reliable evaluation of the QEPAS signal phase shifts and pursue a complete characterization of H_2_S relaxation processes. This type of measurements should require the employment of higher H_2_S concentrations to increase QEPAS SNR and compensate both for background absorption interference arising from the broadening of CH_4_ and H_2_O features, and for Q-factor deterioration.

In addition, our sensor performance, together with the commercial availability of Near-IR sources, demonstrate its applicability for realizing portable safety sensors detecting CH_4_ and H_2_S emissions from natural gas reservoirs or in industrial areas. For this future development, a line-locking configuration will be implemented, rather than full spectral scan acquisitions: H_2_S peak signal would be continuously monitored and, when a non-zero signal indicating a leak arises, the system would send alerts and sequentially look for CH_4_ concentration to i) compensate the H_2_S signal and accurately retrieve its concentration, if needed, ii) evaluate the fire/explosion potential due to the leak. In addition, a future development will be engineering this QEPAS prototype in an explosion proof sensor deployable for in situ leak detection.

## Declaration of Competing Interest

The authors declare the following financial interests/personal relationships which may be considered as potential competing interests: Angelo Sampaolo reports equipment, drugs, or supplies and travel were provided by Polytechnic University of Bari.

## Data Availability

Data will be made available on request.
